# Effects of Motor Learning on Clinical Isokinetic Test Performance in Knee Osteoarthritis Patients

**DOI:** 10.6061/clinics/2017(04)02

**Published:** 2017-04

**Authors:** José Messias Rodrigues-da-Silva, Márcia Uchoa de Rezende, Tânia Carvalho Spada, Lucila da Silva Francisco, Júlia Maria D'Andréa Greve, Emmanuel Gomes Ciolac

**Affiliations:** I São Paulo State University − UNESP, School of Sciences (Campus Bauru), Physical Education Department, Exercise and Chronic Disease Research Laboratory, Bauru, SP, BR; II Instituto de Ortopedia e Traumatologia, Hospital das Clinicas HCFMUSP, Faculdade de Medicina, Universidade de São Paulo, Sao Paulo, SP, BR; III Universidade de Guarulhos − UNG, Guarulhos, SP, BR

**Keywords:** Aging, Isokinetic, Knee, Muscle Strength, Osteoarthritis

## Abstract

**OBJECTIVES:**

To analyze the effects of motor learning on knee extension-flexion isokinetic performance in knee osteoarthritis patients.

**METHODS:**

One hundred and thirty-six middle-aged and older sedentary individuals (111 women, 64.3±9.9 years) with knee osteoarthritis (130 patients with bilateral) and who had never performed isokinetic testing underwent two bilateral knee extension-flexion (concentric-concentric) isokinetic evaluations (5 repetitions) at 60°/sec. The tests were first performed on the dominant leg with 2 min of recovery between test, and following a standardized warm-up that included 3 submaximal isokinetic repetitions. The same procedure was repeated on the non-dominant leg. The peak torque, peak torque adjusted for the body weight, total work, coefficient of variation and agonist/antagonist ratio were compared between tests.

**RESULTS:**

Patients showed significant improvements in test 2 compared to test 1, including higher levels of peak torque, peak torque adjusted for body weight and total work, as well as lower coefficients of variation. The agonist/antagonist relationship did not significantly change between tests. No significant differences were found between the right and left legs for all variables.

**CONCLUSION:**

The results suggest that performing two tests with a short recovery (2 min) between them could be used to reduce motor learning effects on clinical isokinetic testing of the knee joint in knee osteoarthritis patients.

## INTRODUCTION

Osteoarthritis (OA) of the knee is a highly prevalent age-related clinical condition that is associated with joint pain, decreased physical functioning and independence, and loss of muscle strength and power 1-4. Muscle strength plays an important protective role in the progression of knee OA 5-7, and it is inversely associated with physical function in this population 2, 3, 8. Therefore, evaluation of the muscular performance may have important implications to counteract the effects of its reduction on knee OA pathophysiology 2, 3, 7.

The isokinetic dynamometer is the gold standard method for assessing muscular performance, and it provides many clinical variables 9, 10. However, studies analyzing the reliability of isokinetic testing in older subjects have shown intraclass correlation coefficients (ICCs) that vary from 0.29 to 0.99 11-14, suggesting that it may not be reliable in this population. The poor motor performance ability commonly found in older individuals is a possible explanation for the large variation in ICC, which should be considered in isokinetic dynamometry protocols for older subjects 9, 11, 15. Therefore, it has been suggested that the use of a single-session isokinetic testing protocol, as commonly used in young individuals, may not be adequate for older people 11-13.

In this context, performing a familiarization session one day before the main assessment resulted in improved isokinetic testing reproducibility 11. However, in clinical practice, it is not useful to perform a familiarization session on a day that is separate from the main assessment. An alternative would be to use a familiarization session immediately prior to the main testing. Investigating this hypothesis, a study by our group showed that to perform two isokinetic assessments with a short period of recovery between tests (60 sec) improves the performance during the second test, and it should be considered for reducing motor learning effects in postmenopausal women 9.

To the best of our knowledge, no prior study has analyzed the effects of a learning isokinetic test session that was performed immediately before the main testing in knee OA patients. Because joint pain may limit muscle performance in knee OA patients 7, 16, the use of two isokinetic assessments with a short period of recovery between tests may not reduce the motor learning effect, as observed in healthy postmenopausal women 9. Therefore, the aim of this study was to analyze the effects of a familiarization test session, which was performed before the main assessment, on knee isokinetic performance in middle-aged and older patients with knee OA.

## METHODS

### Population and Study Design

The present investigation is a cross-sectional, observational study that was conducted in a single center in Brazil and assessed 136 middle-aged and older patients with an established diagnosis of bilateral (130 patients) or unilateral knee OA for at least one year (Kellgren/Lawrence scale grades of I-IV) 17. The participants had not participated in regular physical activity for at least 1 year (Table 1). All volunteers were recruited from the Osteometabolic Disease Group, Institute of Orthopedics and Traumatology, School of Medicine, University of São Paulo, and they were under drug therapy (diacerein) for the 6 months prior to inclusion.

To meet the eligibility criteria, patients needed to have met the American College of Rheumatology criteria for knee OA 18; not have rheumatoid arthritis or any other rheumatologic disease other than OA; be receiving routine care for OA in the past six months; not have any neurological problems; and be able to understand and agree with the informed consent. Patients with uncontrolled cardiovascular or metabolic disease who underwent surgery or had lower limb injuries during the previous six months were also not included.

After screening, the patients included in the study performed two knee extension-flexion (concentric-concentric) isokinetic tests (Biodex Multi-Joint System 3, Biodex™, Shirley, NY, USA) at 60°/sec (5 reps), with a 2-min interval between tests, for both legs. The isokinetic dynamometer data were then compared between tests. The present study was approved by the Ethics Committee for the Analysis of Research Projects of the *Hospital das Clínicas da Faculdade de Medicina da Universidade de São Paulo* (# 12671). All volunteers read a detailed description of the protocol and provided their written informed consent.

### Isokinetic Evaluation

The knee extension–flexion (concentric–concentric) isokinetic evaluation was performed in the afternoon (between 2:00 and 5:00 p.m.) at a controlled temperature (20–23°C) with a Biodex Multi-Joint System 3 dynamometer (Biodex Medical™, Shirley, NY, USA). Participants were instructed to wear light and flexible clothes, to have a light meal at least 2 hours before the testing, and to refrain from strenuous physical activity for 24 hours before testing. The tests were first performed on the dominant leg. After a standardized warm-up, participants were positioned in the equipment according to the manufacturer's instructions (seated with arms along the body, hands holding the lateral support, and Velcro^®^ belts for stabilization of the trunk, hip and tested limb). Gravitational correction was performed at 40° of knee flexion. Isokinetic knee extension–flexion (concentric–concentric) at 60°/sec was used for data collection. Participants performed three submaximal repetitions prior to data collection. Five maximum repetitions were then performed twice (tests 1 and 2), and a 120 second resting period was used between tests 1 and 2. The same procedure was performed to evaluate the contralateral (non–dominant) leg. The same investigator conducted all isokinetic testing, and verbal stimuli were provided throughout the evaluation. The peak torque (TQ_PEAK_), TQ_PEAK_ adjusted for body weight (TQ_PEAK_/BW), total work, coefficient of variation (CV) and agonist-antagonist (agon/antag) ratio were the assessed variables.

### Statistical Analysis

The Kolmogorov-Smirnov test was used to ensure a Gaussian distribution of the data. Variables are expressed as the mean±standard error of the mean. Two-way ANOVA with repeated measures (leg dominance *vs*. test) was used to indicate a significant difference in the isokinetic variables. The Bonferroni *post hoc* test was used to identify the significant differences that were indicated by two-way ANOVA. The significance level was set at *p*<0.05. The statistical program SPSS™ 17.0 for Windows (SPSS Inc., Chicago, IL, USA) was used to perform the statistical analysis.

## RESULTS

**Figure 1:**
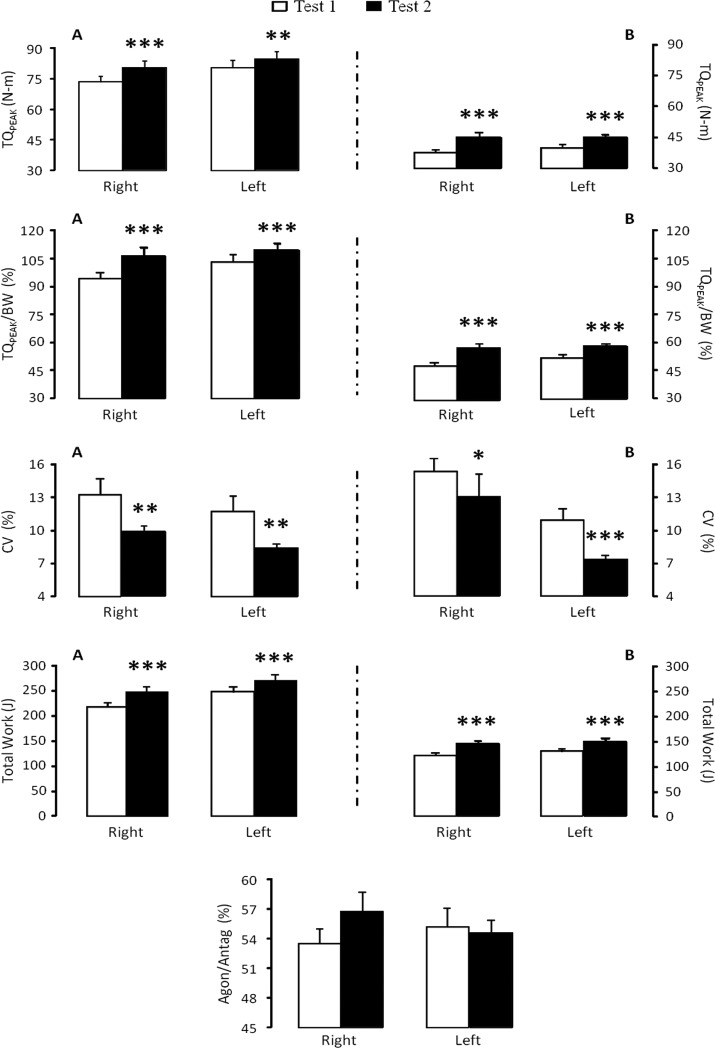
Knee extension (A) and flexion (B) isokinetic data. TQ_PEAK_: peak torque; TQ_PEAK_/BW: peak torque adjusted for body weight; CV: coefficient of variation; and agon/antag: agonist-antagonist ratio. An asterisk denotes a significant difference from Test 1 (*p<0.05; **p<0.01; and ***p<0.001).

The isokinetic evaluation was well tolerated by all patients, and no adverse events occurred during testing. Higher levels of knee extension TQ_PEAK_ (right limb=18.3±5.2%; left limb=9.4±1.2%; *p*<0.01), TQ_PEAK_/BW (right limb=18.3±5.2%; left limb=9.4±1.2%; *p*<0.001), and total work (right limb=20.3±3.6%; left limb=12.2±1.8%; *p*<0.001) as well as lower knee extension CV (right limb=11.6±8.6%; left limb=21.7±8.9%; *p*<0.01) were found in Test 2 (Figure 1). Additionally, there were higher levels of knee flexion (right limb=26.1±5.5%; left limb=14.4±1.8%; *p*<0.001), TQ_PEAK_/BW (right limb=26.1±5.5%; left limb=14.4±1.8%; *p*<0.001), total work (right limb=39.5±11.3%; left limb=25.3±5.8%; *p*<0.001), and lower knee flexion CV (right limb=22.4±12.0%; left limb=10.9±7.0%; *p*<0.05) in Test 2 (Figure 1). The agon/antag ratio did not change significantly between tests. No significant differences were observed between the right and left legs for all variables.

## DISCUSSION

We hypothesized that, because of the limited joint pain-related muscular performance commonly found in knee OA patients 7, 16, the use of two isokinetic assessments with a short period of recovery between tests (2 min) would not have a motor learning effect in the present study. However, the increased knee extension and flexion TQ_PEAK_, TQ_PEAK_/BW, and total work, as well as the lower knee extension and flexion CV observed in Test 2, suggest there was a motor learning effect during the second isokinetic assessment, as observed in healthy postmenopausal women 9. The lack of a difference between the right and left legs for all assessed variables also suggests that leg dominance did not affect motor learning in knee OA patients.

The constant limb velocity and maximum muscle contraction during isokinetic testing provide a direct view of the assessed joint with objective and isolated measures of muscular capacity that may have important implications for training and rehabilitation programming as well as clinical decisions 9, 10, 19. In this context, an increasing number of investigations have assessed isokinetic testing reliability in different clinical populations, including older subjects 11-14, 20, 21, post-polio syndrome 22, and fatigue 23 studies. Specifically, the large ICC variation (0.29 to 0.99) found in older subjects 11-14, 20, 21 suggests that isokinetic testing may not be reliable in this population. An explanation for the large ICC variation found in older subjects may be the poor motor performance ability commonly found in older individuals 9, 11, 15, suggesting that the use of a single-session isokinetic testing protocol may not be adequate for older people 11-13.

In accordance with the above, the role of motor learning in improving muscular performance has been shown in different studies 9, 19,24-27. For example, a familiarization session performed one day before the main assessment improved the isokinetic testing reproducibility in older adults 11. Because performing a familiarization session on a separate day of the main assessment is not useful in clinical practice, we investigated the use of two isokinetic assessments with a short period of recovery between tests (60 sec) in healthy postmenopausal women and found increased knee extension and flexion TQ_PEAK_, TQ_PEAK_/BW, and total work, as well as lower knee extension and flexion CV in the second test 9. The improved isokinetic testing performance found in the present study is thus in accordance with a previous study performed on healthy postmenopausal women. Moreover, the improved performance during the second isokinetic test found in the present study was similar to the improved performance found in healthy postmenopausal women 9, suggesting that commonly observed joint pain caused by cartilage and bone deterioration did not affect motor learning effects in knee OA patients, even with a short period of recovery (2 min) between the isokinetic tests.

One must argue that, although the sample size was large, the number of men was small (N=19, which was nearly 14% of the studied population), which may have affected the results of the present study. However, it is well known that the prevalence/incidence of knee OA is higher in women than men 28. Moreover, the prevalence of women in the total knee arthroplasty waiting list at the Institute of Orthopedics and Traumatology, School of Medicine, University of São Paulo is nearly 70% (data not published). Therefore, the lower number of men in the present study is representative of the sex differences in knee OA prevalence/incidence.

The present study has some limitations that should be acknowledged. Unlike a previous study on healthy postmenopausal women that included 1 min of recovery between tests 9, we adopted a larger recovery period between tests (2 min) with the purpose of alleviating a possible sensation of pain felt by knee OA patients. However, we do not know if this recovery range between tests is the most appropriate. The present population was tested at a single angular velocity (60°/sec), and we do not know if the motor learning effects found in the present study would occur in a similar way with lower or higher angular velocities. Therefore, future studies are needed to clarify these issues.

Muscular capacity may play an important protective role in the progression of knee OA, and it is strongly associated with the physical function in knee OA patients 2, 3, 5, 7. Therefore, a muscular capacity assessment is an important tool for clinical making decision in this population. For example, the objective and isolated measures of muscular capacity assessed by isokinetic testing allows for a more judicious rehabilitation program to be conducted 9. Moreover, isokinetic testing may detect deficiencies in the balance between flexor and extensor muscles of the knee (a balance that is required for uniform gait and postural balance), which may help in the design of specific rehabilitation exercise programs according to the patient's needs 19. In this context, the results of the present study have important clinical implications.

In summary, isokinetic testing performance (TQ_PEAK_, TQ_PEAK_/BW, total work, and CV) was improved in the second knee extension-flexion evaluation. This result suggests that performing two tests with a short recovery (2 min) between them could be used to reduce the motor learning effects on clinical isokinetic testing of the knee joint in knee OA patients.

## AUTHOR CONTRIBUTIONS

Rodrigues da Silva JM participated in the study design, data collection, analysis and manuscript preparation. Rezende MU participated in the study design and manuscript preparation. Spada TC and Francisco LS participated in the data collection and manuscript preparation. Greve JM participated in the study design and manuscript preparation. Ciolac EG participated in the study design, data analysis and manuscript preparation.

## Figures and Tables

**Table 1:** Baseline patient characteristics.

Variable	Patients
N	136
F/M	117/19
Age (years)	64.3±0.8
Body weight (kg)	78±16.2
Height (m)	158±0.09
BMI (kg/m^2^)	31.9±0.5
Bilateral osteoarthritis (N)	130
Unilateral osteoarthritis R/L (N)	2/4
Kellgren and Lawrence scale	
Degree I (N (%))	4 (2.7)
Degree II (N (%))	36 (25.3)
Degree III (N (%))	58 (43.3)
Degree IV (N (%))	38 (28.7)

N: number of patients; F: female; M: male; BMI: body mass index; R: right; and L: left
